# Notes and News

**Published:** 1892-01-16

**Authors:** 


### Notes and News.

Collected and Communicated.

The Boscombe Hospital, always alert and active, has already spread the wing, the foundation of which was so recently laid, and its usefulness is in full flight. The new improvements, which were absolute necessities, consist of a comfortable out-patients' room, a dispensary, bath-rooms, an operating-room—for which, however, the operating-table is still wanted—and two pleasant rooms for the Matron. Equipped with these long-needed additions, this bright little hospital sets sail, under command of its deservedly popular Matron and House Surgeon—Miss Thomassen and Dr. Weary—down the tide of the New Year upon which we have just entered.

We have had the disagreeable task of quoting articles lately from one source unfavourable to our London hospitals. In the *British Medical Journal* for January 9th, we find for them a fair and able defence, which after a preliminary paragraph runs as follows: "There is, or ought to be a very great difference between zeal to detect and make war upon 'Mismanagement and corruption,' and readiness to affix such a stigma upon institutions well managed, beneficent, and controlled by persons whose motives are pure, whose acts are public spirited, self-devoted, and highly efficient. We do not think, however, that the distinction is adequately observed in some recent journalistic utterances, in which such words are freely used, and such imputations conveyed on the management of S. Bartholomew's and the London Hospital. If there were the faintest shadow of foundation for the just use of such language, or for bringing such charges, we should be the last to blame any who might have the courage to perform a painful public duty. As it is, we make bold to say, that the charge has been made recklessly or ignorantly, and as the merely parrot-like iteration of some catching headline of an irresponsible paragraphist, it cannot but be hurtful, as it is most unjust to the really able and good men who preside over the affairs of these most admirable and beneficent institutions, and what is worse, it is mischievous to the general cause of hospital charity, which peculiarly deserves, or needs at this time ample and even munificent support to enable them to fulfil their great self-imposed tasks. There may be, and have been, questions of administration which lead to differences of opinion, and indeed, it would be impossible in such great establishments, any more than in many small ones, that such questions and occasions for criticism should not arise. But we only assert what every rational person acquainted with the facts knows, and must confirm, when we say that the London Hospital, to take the stronger case first, is one of the most admirably conducted, and one of the most beneficent institutions which exists even in London, the greatest centre of public charity which the world has ever seen, and that even to hint at scandal or corruption in such a connection, is to betray a very imperfect sense of public responsibility, and to use a very heated and unsuited vocabulary."

A very interesting ceremony took place on Sunday, the 20th ult., in connection with the Home and Hospital for Jewish Incurables. This was the consecration of a synagogue in the grounds of the Home. The edifice was erected at the expense of Mr. Michael Zeffert, and presented by him to the institution in memory of his mother. The building has accommodation for 85 persons. The consecration ceremony was conducted by the Chief Rabbi, who delivered a most touching and eloquent address at the conclusion of the service. In this, he remarked that he, himself, objected to the title of "incurable," as applied to the Home, considering the term implied a want of faith; and neither was it a strictly scientific expression, as the advance in medical science was such that fewer and fewer cases could be declared absolutely incurable. The Rabbi appealed earnestly for increased support for the Home, which has been now established for two years. Many friends of the Home contributed gifts towards the furnishing of the synagogue, which is a very complete and tasteful little edifice.

Evidence of the good-will of those who are too often the involuntary recipients of the benefits conferred by hospitals was given in a substantial form on Friday night at the little hospital in the Royal Victoria and Albert Docks, which is one of the branches of the Seamen's Hospital Society. The occasion was the handing over to the Society a sum of £216, which had been collected in the neighbourhood in the incredibly short space of time of two months. Mr. F. T. Tucker, the Superintendent of the Railway Department of the Docks, to whom too much praise cannot be given for the way in which he has worked for the hospital, occupied the chair. Mr. Thomas, one of the Hon. Secretaries, to whom the success of the meeting was greatly due, read a list of the subscribers. From this it appeared that an entertainment in the Albert Hall, Canning Town, had brought in £60, and the balance had been collected by the employés of the Docks, shipping firms, and other works in the neighbourhood. Over £200 is probably the largest sum ever collected in a similar manner for a hospital. The institution, though primary for sailors, is opened day and night, and will receive at any time landsmen suffering from accidents or emergencies. The hospital has only been opened 18 months, and as evidence of the needs of its existence over 500 in-patients and 2,000 out-patients were treated last year. Previous to the establishment of this branch of the Seamen's Hospital Society serious cases of accident had to be conveyed, often with much danger and suffering to the patients, a distance of two or more miles.

A very bright and pretty entertainment for patients past and present, as well as nurses, took place at the London Homœopathic Hospital, on Thursday, the 7th inst. A ward now in disuse was appropriated by stage and audience, and some very pretty tableaux were shown to an appreciative company. The *dramatis personæ* consisted of medical staff, nurses, and patients (children). Nurses Dora and Geraldine formed a charming picture as ministering angels. A voice behind the scenes gave a pleasing rendering of "Angels Ever Bright and Fair." During the several scenes in which King Winceslas was represented, a choir of nurses gave the well-known refrains. Perhaps the most popular tableau was the one in which the celebrated "Sarey Gamp" appeared in charge of her unfortunate patient. This scene was of a more dramatic nature than former ones in order to give "Sarey" full opportunities of exhibiting her talents to an interested audience. Her efforts were received with shouts of laughter by the children, and doubtless with a feeling of amused superiority by the nurses. At the conclusion of this part of the entertainment a Christmas tree for the children and presents for the nurses were prepared in the children's ward. Amongst the nurses present at the entertainment was the Sister who attended Count Gleichen in his last illness. We were much struck with the courteous manners of the nurses whom, as a complete stranger, we came in contact with. At the Homœopathic they evidently believe that "manners makyth man, and woman as well.

